# Editorial: RNAi Based Pesticides

**DOI:** 10.3389/fpls.2021.714116

**Published:** 2021-07-29

**Authors:** András Székács, Azeddine Si Ammour, Michael L. Mendelsohn

**Affiliations:** ^1^Agro-Environmental Research Centre, Institute of Environmental Sciences, Hungarian University of Agriculture and Life Sciences, Budapest, Hungary; ^2^Fondazione Edmund Mach, San Michele all'Adige, Italy; ^3^United States Environmental Protection Agency, Washington, DC, United States

**Keywords:** double stranded RNA, RNA interference, pest control, regulation, gene silencing, environmental risk assessment, non-target organisms

Development of new pesticide tools for farmers is needed to help increase food production. Approaches based on the use of nucleic acids triggering RNA silencing in plant pathogens and other pests in a sequence-specific manner are very promising and few preliminary works reported efficient protection of crops. Topically applied double stranded ribonucleic acids (dsRNAs)/small interfering ribonucleic acids (siRNAs) or spray-induced gene silencing (SIGS), also termed non-transformative RNAi technology to differentiate it from genetically modified (GM) plants designed to induce gene silencing through RNAi, are of particular interest.

On April 10-12, 2019, the Conference on RNAi Based Pesticides, held in Paris, France, supported by the Organisation for Economic Co-operation and Development (OECD)'s Co-operative Research Programme on Biological Resource Management for Sustainable Agricultural Systems, brought together academic and industry researchers, risk assessment experts, and environmental and food safety regulators, to discuss current research and policy issues related to this newly emerging, potential crop protection technology. The conference was attended by representatives from 14 OECD countries (Australia, Austria, Belgium, Canada, the Czech Republic, Denmark, Estonia, France, Germany, Hungary, the Netherlands, Switzerland, the United Kingdom, and the United States of America), and the European Food Safety Authority (EFSA). Gathering such a broad range of experts together allowed in-depth exploration of the possibilities of the application of external RNAs as pesticide active ingredients and discussions on the current state of knowledge and topics to help in developing considerations for risk assessment and corresponding foreseen government regulations. Two major questions addressed during discussions are (1) Are the current approaches to environmental and human health risk assessment of conventional and biological pesticides and or GM technologies applicable to the risk assessment of dsRNA based pesticides? and (2) Are additional data needed to be developed for dsRNA based pesticides?

On the basis of the conference presentations and expert discussions at the event, the present collection of research papers was launched as a joint Research Topic of the leading scientific periodicals, *Frontiers in Plant Science* and *Frontiers in Microbiology* to publish studies presented at the conference and submissions from experts working in the subject. Overall, 50 authors contributed 14 articles (2 original research articles, 5 reviews, 3 mini-reviews, and 4 perspective papers) discussing the possible utilization, but also rigorously considering the possible hazards and risks of external application of dsRNA molecules, their environmental fate, and effects possibly exerted on non-target organisms and human health.

## The Mode of Action of RNA Based Pesticides

Several papers discuss the molecular biological mechanisms and other fundamental aspects of RNA-silencing. In his introductory overview on the mode of action of dsRNA induced sequence-specific RNA-silencing as a defense pathway in invertebrates and plants against viruses that produce dsRNA, Svoboda describes the main steps in the RNA degradation mediated by core protein components RNase III Dicer producing siRNA duplexes from dsRNA, endonuclease Argonaute, and RNA-dependent RNA polymerase. Besides outlining the molecular mechanism, he addresses the RNAi technology as a potent tool against agricultural pests but also warns about unintended off-target effects due to RNAi activity in species other than the pest organism aimed, about the possibility of transfer of small RNAs and RNAi among species and about the potential emergence of resistance to RNAi.

A detailed analysis of enzymes Argonaute and Dicer in a model grass plant the purple false brome (*Brachypodium distachyon*) is presented by Šečić et al.. Previously, various forms of the two enzyme families have been identified in the most common model organism in plant biology, the thale cress (*Arabidopsis thaliana*). This report identifies an expanded range, 16 members of the Argonaute family and 9 members of the Dicer family in *B. distachyon*, and also provides domain characterization, phylogenetic investigation supported by 3D protein modeling, as well as organ- or tissue-specific expression analysis of these proteins.

## Pesticide Environmental Risk Assessment and Management

In most countries of the world, environmental risk assessment in the pesticide registration process is supported by non-target organism toxicity and environmental fate laboratory testing and, in some cases, field studies. Predicted environmental exposure concentrations (PECs) of the target compound(s) must not exceed given thresholds considered safe for non-target organisms, where these exist. Processes may differ internationally from region to region.

Pesticide registration in the European Union (EU) is completed in a dual process: the active ingredients are approved at EU level, and the formulated plant protection products are authorized at Member State levels. In the United States (US), the Environmental Protection Agency (EPA) requires registration of pesticide products and evaluates the pesticide active ingredient and formulated products. States or territories within the US require pesticide registration as well.

## Application Possibilities of RNA Based Pesticides

The fundamental biochemical study by Šečić et al., mentioned above, in a model grass plant opens a knowledge base toward agronomically important cereals targeted by RNAi-based plant protection strategies e.g., barley or wheat. In addition, several other studies in the Research Topic mention various crop application promises. A direct possibility of application of external RNAi in crop protection is to spray dsRNA directed against pest-specific genes onto plants. Fungi or insects will take up these RNAs and process them to complex siRNA mixtures, which affect their survival or growth. Aspects of practical applications either against insect or plant pathogen microorganism pests from RNA delivery in water-soluble formulations (including foliar applications, trunk injection, and substance administration via irrigation) are reviewed by Cagliari et al. also summarizing successful application cases so far. The authors also compare the advantages of transformative (i.e., transgenic) and non-transformative (i.e., spray) RNAi applications.

Werner et al. illustrate RNAi applicability by assessing the efficacy of dsRNAs applied in SIGS in barley to suppress infestation by *Fusarium graminearum*, and verify effective gene silencing by measuring declines in the transcript levels of target genes in *F. graminearum* grown in the infected leaf tissue of the plant treated with different targeting dsRNA constructs. Interestingly, better RNAi effects were reached when manually designed dsRNA constructs (40–74% inhibition of gene expression) were used than in the case of computationally-designed constructs (44–52% inhibition of gene expression).

Different delivery strategies of RNAi-based products (i.e., dsRNA) for insect control are addressed by Christiaens et al.. They draw distinctions among host-induced, virus-induced, and spray-induced gene silencing (HIGS, VIGS, and SIGS). This review also summarizes field application difficulties e.g., physiological and cellular barriers leading to efficacy loss in insects, and advises novel non-transgenic delivery technologies e.g., polymer or liposomic nanoparticles, peptide-based delivery vehicles, and viral-like particles to overcome these barriers.

## Risk Assessment of RNA Based Pesticides

RNAi can be utilized in crop protection either by plant-incorporated protectants through plant transformation (i.e., transgenic plants) or by non-transformative strategies through SIGS. Prior to the consideration of externally applied dsRNAs, GM plants designed to induce gene silencing through RNAi have already been developed and submitted as intended regulated products for authorization including food/feed safety assessment and environmental risk assessment. EFSA has already held an international scientific workshop and commissioned three external scientific reports on the subject, published an internal note on the strategy of off-target identification/prediction and risk assessment of RNAi based GM plants, as presented by Papadopoulou et al.. EFSA generally considers existing science-based risk assessment approaches for GM crops suitable also for RNAi-based GM plants, with certain specificities in the latter group. Another special form of transformative RNAi application that utilizes microbe- or virus-induced gene silencing also requires in-depth environmental assessment and falls under the regulation of GM organisms, as such methods involve release into the environment of viruses, bacteria, yeasts, or fungi genetically modified to act as a vector to generate RNAi induction by a continuous production of dsRNA into the host. Environmental decay or inactivation of the GM microbe- or virus vectors after an application is an issue in this field. The external application of naked or formulated dsRNAs does not imply this aspect, as it does not involve the release of a live reproducing organism.

Bioinformatic analyses provide useful information for risk assessment, as non-target organisms with genes with some level of sequence homology to the gene intended for silencing in the target pest/pathogen can be identified by them, but the presence of RNAi activity cannot be reliably predicted in all representative non-target organisms, therefore, this approach cannot be used as a stand-alone tool. On the basis of the assessment of the above GM plants, Rodrigues and Petrick are of the opinion that the experience with the review of dsRNA-based GM crops has demonstrated that the existing regulatory paradigm for biologically based crop protection products is also adequate for the mode of action of externally applied dsRNA.

Environmental stability is a key issue in the use of dsRNAs. Bachman et al. emphasize that in order to provide environmental risk assessment and information on potential exposures, *in planta* produced (GM crops) and topically applied dsRNA (spray application), dsRNA must be successfully measured in relevant environmental compartments (soil, sediment, surface water). Unformulated dsRNAs were found to be highly labile (reported DT_50_ are in the range of 0.5–0.7 days), decomposed mostly by rapid microbial degradation of nucleic acids, but photodegradation and wash-off due to rain or dew also contribute to dissipation. Formulations can enhance dsRNA stability in the environment and can facilitate penetration through physical or biochemical barriers in target pests. Thus, formulations of dsRNA with layered double hydroxide nanosheets (“BioClay”) or a shaped poly(2-(dimethylamino)ethyl acrylate) analog could achieve a 4-fold increase in its stability. BioClay formulation of dsRNAs is discussed in more detail by Fletcher et al..

Formulation impacts risk assessment for topically applied dsRNA, since certain formulants can substantially increase persistence. Exposure and hazard levels, therefore, have to be estimated considering the formulation, only taking the short half-life of the naked siRNA into account is not sufficient and would obviously be misleading. Therefore, information and/or studies on the impact on uptake and environmental persistence that the formulation presents are important to characterize exposure to the dsRNA. Where specific product formulations impact barriers to and uptake of the dsRNA, product-specific formulation toxicology testing on organisms or test surrogates would help better characterize the potential for hazard. The necessity of such testing depends on the legal requirements in the different OECD member countries as well as the characterization of the product.

Among the assessments presented in this Research Topic Fletcher et al., Neumeier and Meister, and Rodrigues and Petrick give utterance to the view that miRNA-like off-target activity of externally applied dsRNAs on various species (other than the pest) is negligible. Rodrigues and Petrick conceive that on the basis of exposures through different routes (ingestion, dermal absorption, inhalation), possible biological barriers, and the history of safe RNA consumption considered, harmful effects to humans are unlikely at dietary uptake level.

Oppert and Perkin propose a genome-wide expression analysis, termed RNAiSeq, in targeted pests (insects) to validate effective knockdown of target genes and to assess effects of possible knockdown on non-target genes by RNAi. Using this method on a coleopteran model insect the red flour beetle (*Tribolium castaneum*) they validated effective knockdown of various genes in several case studies e.g., a gene (*TC01101*) encoding the primary cysteine peptidase, a major digestive enzyme in *T. castaneum* larvae, and other genes encoding enzymes or other proteins involved in cuticle physiology.

In contrast, Raybould and Burns state that the establishment of any off-target effect inventory produced by profiling methods is unnecessary as such a fundamental research approach does not effectively support decision-making. The risk assessor only needs to take into consideration whether a dsRNA based agent presents acceptable or unacceptable risks. In their opinion methods for assessing exposure of and toxicity to non-target organisms by dsRNA based substances can be adapted from those being currently applied for chemical pesticides. They urge the use of targeted risk assessment on the basis of thresholds of unacceptability. If given externally applied dsRNAs do not pose unacceptable risk i.e., their toxicity:exposure ratio does not exceed a pre-set level, they should be approved. Romeis and Widmer allude to a somewhat similar approach, yet acknowledging the need for protection of certain non-target organisms in the agroecosystem, for example, those representing biodiversity protection goals of valued ecosystem services (Millennium Ecosystem Assessment [MEA], 2005). They consider an environmental risk assessment approach similar to that used in the case-by-case assessment of GM plants, but allowing flexibility to non-target risk assessment and being based on the selection of the most appropriate negative and positive control treatments suitable for externally applied RNAi based pesticides. In addition, they also emphasize the importance of the formulation in which dsRNAs are being applied (see above).

Strategies including RNA modifications or pooling of siRNAs to reduce off-target effects are outlined by Neumeier and Meister. Chemical modification (2′-*O*-methylation or incorporation of a locked nucleic acid) on the siRNA guide strands are reported capable of weakening the interaction between the guide strand and the target, and therefore, reducing miRNA-like off-target activities, without limiting siRNAs typically fully complementary to their on-target. Pooling at very low concentrations of multiple siRNAs directed against the same on-target at different positions but with different off-target signatures has also been indicated to reduce miRNA-like off-target effects.

## Expert Discussions on the Topic

Views and opinions expressed on the environmental fate of dsRNAs, as well as risk assessment on non-target organisms and human health during panel and overall discussions at the conference are summarized by Mendelsohn et al.. Key considerations from these conference discussions have already been incorporated into an OECD Working Document [OECD (Organisation for Economic Co-operation and Development), 2020] that facilitates regulators in evaluating externally applied dsRNA based products for potential environmental risks. Thus, diverse discourses regarding the use of dsRNA products in agriculture, regulatory, and risk assessment experience with dsRNA based products, and focused thematic issues, representing multiple perspectives, are reported. A definite intention of all scientific events sponsored by OECD CRP is to particularly address policy issues to help decision-makers to formulate official regulations better serve to support sustainable agriculture objectives. Therefore, such policy relevance has strongly been emphasized in the conference discussions. A consensus has been reached that current protocols used in hazard and risk assessment of pesticides have to be tailored for dsRNAs. Additionally, any evaluation of a dsRNA-based pesticide should include monitoring for degradation of the dsRNA over time. The importance of product formulation on environmental persistence of dsRNA and uptake by non-target organisms have been emphasized as topics that require special consideration. In addition, although health risks on humans and other mammals to environmental dsRNAs were deemed to possibly extendable to other vertebrates, it has been emphasized that current knowledge is insufficient to predict corresponding responsiveness across invertebrate taxa. For organisms that have been demonstrated to be responsive to environmental RNA, consideration of life cycle studies (growth, development, and reproduction) and studies on other non-lethal effects could be considered. A lacking thematic issue, unfortunately not substantially addressed during the conference, is the possibility of pest resistance development. We understand that this issue may also be considered.

## Author Contributions

AS and MM were an organizer and a section mediator at the OECD Conference on RNAi Based Pesticides and edited on behalf of the OECD conference contributions to this Research Topic. All authors were involved in writing this editorial and agree to its final version.

## Author Disclaimer

The opinions expressed in this paper are the sole responsibility of the authors and do not necessarily reflect those of the OECD or the governments of its Member countries.

## Conflict of Interest

The authors declare that the research was conducted in the absence of any commercial or financial relationships that could be construed as a potential conflict of interest.

## Publisher's Note

All claims expressed in this article are solely those of the authors and do not necessarily represent those of their affiliated organizations, or those of the publisher, the editors and the reviewers. Any product that may be evaluated in this article, or claim that may be made by its manufacturer, is not guaranteed or endorsed by the publisher.
